# Epidemiological characteristics of *SHV, cmlv,* and *FosA6*-producing carbapenem-resistant *Klebsiella pneumoniae* based on whole genome sequences in Jiangsu, China

**DOI:** 10.3389/fmicb.2023.1219733

**Published:** 2023-07-19

**Authors:** Jiaying Zhu, Yanmin Ju, Xinyu Zhou, Taoyu Chen, Xiangkai Zhuge, Jianjun Dai

**Affiliations:** ^1^College of Pharmacy, China Pharmaceutical University, Nanjing, China; ^2^Department of Nutrition and Food Hygiene, School of Public Health, Nantong University, Nantong, Jiangsu, China; ^3^Department of Orthopedics, The First Affiliated Hospital of Baotou Medical College, Inner Mongolia University of Science and Technology, Baotou, China; ^4^MOE Joint International Research Laboratory of Animal Health and Food Safety, Key Laboratory of Animal Bacteriology, Ministry of Agriculture, College of Veterinary Medicine, Nanjing Agricultural University, Nanjing, China

**Keywords:** CR-hvKPs, *KPC-2*, pHN7A8, *bla*_CTX-M-65_, *OXA-10*, Fe_2_C

## Abstract

Carbapenem-resistant *Klebsiella pneumoniae* (CRKP), particularly those with high virulence, cause invasive disease in clinical settings. An epidemiological investigation was conducted on the evolution, virulence, and antimicrobial resistance of CRKP isolates in two tertiary teaching hospitals in Jiangsu, China from November 2020 to December 2021. There were 31 different CRKP strains discovered. We performed whole genome sequencing (WGS) on 13 *SHV, cmlv,* and *FosA6*-producing CRKP to reveal molecular characteristics. Five ST15/ST11 isolates had CRISPR-Cas systems. By conjugation tests, *KPC-2* can be transmitted horizontally to *E. coil*. A conjugative pHN7A8-related multi-resistance plasmid (*KPC-2*, *bla*_CTX-M-65_, *bla*_TEM-1_, *fosA3*, *catII*, and *rmtB*) was first discovered in CRKP clinical isolates. Using bacteriological testing, a serum killing assay, and an infection model with *Galleria mellonella*, three ST11-K64 *KPC-2* generating carbapenem-resistant hypervirulent *Klebsiella pneumoniae* (CR-hvKP) were identified. These strains harbored a virulent plasmid and an IncFII-family pKPC/pHN7A8 conjugative plasmid, which led to hypervirulence and resistance. One of these CR-hvKPs, which co-harbored *KPC-2*, *NDM-6*, *SHV-182*, *SHV-64,* and *bla*_CTX-M-122_ genes, was first discovered. Importantly, this CR-hvKP strain also produced biofilm and had non-inferior fitness. The widespread use of ceftazidime/avibactam might provide this CR-hvKP with a selective advantage; hence, immediate action is required to stop its dissemination. Another important finding is the novel ST6136 in *K. pneumoniae*. Finally, the sterilization efficiency rates of Fe_2_C nanoparticles in CRKP were more than 98%. Furthermore, our novel antibacterial Fe_2_C nanoparticles may also provide a therapeutic strategy for infections.

## Introduction

Antimicrobial resistance in bacteria is a worldwide threat to public health that endanger the advancement of various medical fields. In particular, carbapenem resistance is of concern since these agents are often the last line of treatment due to the multidrug-resistant (MDR) Gram-negative bacteria. The World Health Organization recognizes carbapenem-resistant *Enterobacterales* (CRE) as being among the highest priority pathogens. The most prevalent bacterial species among the CRE is carbapenem-resistant *Klebsiella pneumoniae* (CRKP). A total of 70–90% of clinical CRE infections in the European Union and China are caused by CRKP (Grundmann et al., 2017; Zhang et al., 2017). A longitudinal large-scale CRE study from 65 hospitals across China reported that predominant *KPC*-ST11 *K. pneumoniae* exhibits extensive resistance to most available antibiotics (Wang et al., 2018). Moreover, these CRKP isolates have a significant risk of morbidity and mortality and are resistant to the majority of current medicines (Rossi et al., 2021). *K. pneumoniae* was shown to be the second-leading respiratory infection among COVID-19 patients in a study that was carried out in China (Zhu X. et al., 2020). A percentage of individuals may experience complications from coronavirus disease due to bacterial and fungal co-infections (COVID-19; Rawson et al., 2020).

First reported in Taiwan, a novel hypervirulent *K. pneumoniae* (HvKP) strain causes invasive pyogenic liver abscesses (Siu et al., 2011). The number of HvKP infections increased over the past three decades in parts of Asia. As such, the region with the most prevalent infections is Asia, with K1 and K2 representing the most common serotypes (Siu et al., 2012; Russo et al., 2018). Particularly in locations where HvKP is widespread, HvKp infections have the potential to significantly complicate the course of COVID-19 (Hosoda et al., 2021). Moreover, the MDR and HvKP strains developed independently in different clonal groups (Chen et al., 2020). But CR-hvKP has recently emerged as a significant entity in China (Zhang et al., 2020). Previous studies have reported several mechanisms for widespread CR-hvKP (Gu et al., 2018; Chen et al., 2020; Tian et al., 2022). The emergence of these strains further complicates clinical practice as these strains are concurrently highly contagious, multidrug-resistant, and hypervirulent. Therefore, these strains ought to be thought of as superbugs since they can seriously endanger public health (Gu et al., 2018). This emergence has already been reported in China, Brazil, and the United Kingdom (Andrade et al., 2014; Lee et al., 2017; Roulston et al., 2018); however, the state of the epidemic and transmission mechanisms are poorly understood. To identify, monitor, and implement interventions that will reduce the potential threat that such infections may do to human health, a deeper comprehension of how bacteria evolve to become more dangerous is necessary. The current research aimed to provide information on the molecular epidemiology of CRKP and CR-hvKP in two tertiary teaching hospitals in Jiangsu Province during a one-year period.

## Materials and methods

### Bacterial strains and clinical data

Patients with CRKP infections were retrospectively included for this longitudinal, molecular epidemiological investigation at two tertiary teaching hospitals in Jiangsu province. Two carbapenem-sensitive strains were included as controls. A clinical strain was considered CRKP if it is resistant to one of the following three carbapenems (imipenem, meropenem, or ertapenem). All isolates were identified by the VITEK-2 compact system (BioMerieux Italia S.p.A). We gathered and analyzed clinical information, such as demographics, clinical diagnoses, specimen type, and sample collection date, from patients who had CRKP infections.

### Antimicrobial susceptibility testing

According to the guidelines of CLSI (M100, 2021), antimicrobial susceptibility testing was done using agar and broth microdilution techniques. The results were interpreted following the CLSI (M100, 2021). The breakpoint of tigecycline was interpreted based on FDA guidelines.

### Microbiological features

According to the CLSI M100-S31 recommendations, the Modified Carbapenem Inactivation Method (mCIM) test and ethylenediaminetetraacetic acid-Modified Carbapenem Inactivation Method (eCIM) test were used to detect the phenotype of carbapenemase. Polymerase chain reaction (PCR) and sequencing were used to confirm resistance genes (Zhu J. et al., 2020). We also screened for virulence genes, including aerobactin (*iucA*), salmochelin (*iroN*), and regulator of the mucoid phenotype (*rmpA* and *rmpA2*), as previously described (Zhang et al., 2020).

### Sequence types (STs) and serotype analysis

Multilocus sequence typing (MLST)^1^ was used to determine STs. The *wzi* gene, whose sequences were matched with *wzi* databases (see text footnote 1), was used to identify the serotypes.

### Virulence assay

A string test was used to detect the hypermucoviscous phenotype (Yang et al., 2022) as previously described (Gu et al., 2018).

CR-hvKp was defined by the positive combination of an *in vitro* and *in vivo* virulence assay. We next focused on the virulence phenotypic characterization of the three isolates that tested positive for all four virulence genes. A strain without all four virulence genes was used as the negative control. According to prior studies, a serum killing test was performed to assess *in vitro* virulence (Abate et al., 2012; Zhang et al., 2020). A *G. mellonella* larvae infection model was also performed on isolates with all four virulence genes. Healthy larvae weighing between 250 and 300 mg. Phosphate buffered saline (PBS) was used in the blank control groups. Using a micro-sample syringe, 10 μL of bacterial suspension (10^7^ CFU/mL) was injected into each larva’s left proleg. Each group contained 10 larvae. The larvae mortality rates were observed every 12 h for 3 days.

A biofilm formation assay was performed as previously described (Liu et al., 2022). A 10 μL bacterial suspension (0.5 McFarland standards) and 190 μL LB broth were incubated for 36 h at 37°C in 96-well plates. The OD 570 nm was measured using a microplate reader (Tecan). The results were interpreted as described in previous studies (Liu et al., 2022). At least three above tests were conducted on each strain.

### Fitness analysis

An assay that measures growth curves was used to assess fitness. Both the one CRKP-16 isolate and three CR-hvKP isolates were chosen. Overnight cultures were vigorously aerated and diluted to OD_600_ = 0.01 before being cultivated at 37°C (shake at 200 rpm). Every hour, the OD_600_ was used to measure the culture cell density.

### Whole genome sequencing (WGS)

Thirteen strains with *SHV*, *FosA6*, and *cmlv* were sequenced utilizing the HiSeq sequencing platform. Seven of them were also analyzed using PacBio Sequencing Technology (PacBio). The CRKP genome was sequenced using a combination of the PacBio RS/HiSeq sequencing platform and Illumina sequencing platforms.

The whole genome data were entered into the NCBI database under BioProject number PRJNA892271. The virulence and antimicrobial resistance genes were downloaded from the Virulence Factor Database (VFDB) and Comprehensive Antibiotic Resistance Database (CARD), respectively. Based on the maximum likelihood in MEGA 11, the phylogenetic tree was created using the MLST of 31 strains. Another phylogenetic tree consisting of 78 strains was constructed using PhyML v3.0 (randomized accelerated maximum likelihood, the results corrected with bayes) with 100 bootstrap repetitions. The Interactive Tree of Life web tool^2^ was used to display and annotate the generated tree.

### Plasmid analysis

By checking all accessory genes against a plasmid database, all genes connected to plasmids were downloaded. The plasmid-related contigs were extracted from the genomes and subjected to a BLASTN analysis against known plasmids in the GenBank database with a threshold of 80% identity for contigs <10 kb and 80% identity for contigs >10 kb. The assembly of related sequences produced representative contigs with 90% coverage. By querying against the plasmid database, we selected the three most similar plasmids for comparative genomic analysis. Plasmid circular structural maps were generated using CGView software. Plasmid sequences were also analyzed using Plasmid-Finder version 2.1 and oriTfinder.

### Conjugation assay

*E. coli* EC600 was used as the recipient of conjugation assays to assess the five WGS CRKP plasmids’ transferability. The CRKP-7, 16, 17, 19, and 21 isolates were used as donors. Each of the donor and recipient bacteria were cultured in fresh Luria Bertani (LB) broth at 37°C to reach the logarithmic phase. The mixture of the donor and recipient isolates was mixed in a 2:1 ratio (donor/ recipient) and spotted on 0.45 μm membrane filters on LB plates. After 12 h of incubation at 37°C, the conjugates were spread on LB agar with 2 μg/mL imipenem and 32 μg/mL rifampin. Transconjugants were identified by PCR assay and minimum inhibitory concentration (MIC) profiles.

### Antibacterial evaluation of Fe_2_C nanoparticles *in vitro*

To date, the development of new antibiotics for CRKP with clear antibacterial mechanisms remains a significant challenge. Herein, novel alternative antibacterial Fe_2_C nanoparticles (NPs) with peroxidase-like activity was synthesized by high performance liquid chromatography according to our previous report (Sun et al., 2021). Carbapenem-sensitive *K. pneumoniae* (CSKP)-34, CRKP-16, and CR-hvKP-7 were diluted with fresh liquid LB medium. Following that, the bacterial samples were separated into three categories, including (a) control, (b) Fe_2_C + glucose 5 μL + glucose oxidase 1 μL, and (c) glucose 5 μL. The final iroN concentration of Fe_2_C was 75 μg/mL. The total volume of the solution was 400 μL. A hundred microlitre of the bacterium diluent (1:10^5^ dilution with saline) was put onto the LB agar plate after 3 h of incubation, and it was then cultivated at 37°C for 12 h.

## Results

### Clinical characteristics

In this study, 31 CRKP isolates were collected from two hospitals between November 2020 and December 2021. The CSKP-33 and CSKP-34 isolates were sensitive to carbapenems and were included as control strains. Patients with CRKP were 66 years old on average (Supplementary Table S2). The majority of cases (15, 48.39%) were admitted into the intensive care unit (ICU). Most sites of clinical samples were sputum (13, 41.94%), followed by blood and urine (6, 19.35%, respectively). There were 18 males (58.06%) (Supplementary Table S2).

### Antimicrobial susceptibility and genetic characteristics

The CRKP isolates had a high degree of resistance to imipenem (30, 96.77%), ertapenem (30, 96.77%), and meropenem (28, 90.32%). There were also similar antimicrobial susceptibility profiles, all of them were sensitive to colistin (31, 100%), while being resistant to the majority of the tested antibiotics. The susceptibility of amikacin was 41.94% (*n* = 13). Four tigecycline-resistant and five tigecycline-intermediate strains were identified and the MICs of tigecycline for these strains were 4–16 mg/L (Supplementary Table S1).

**Figure 1:**
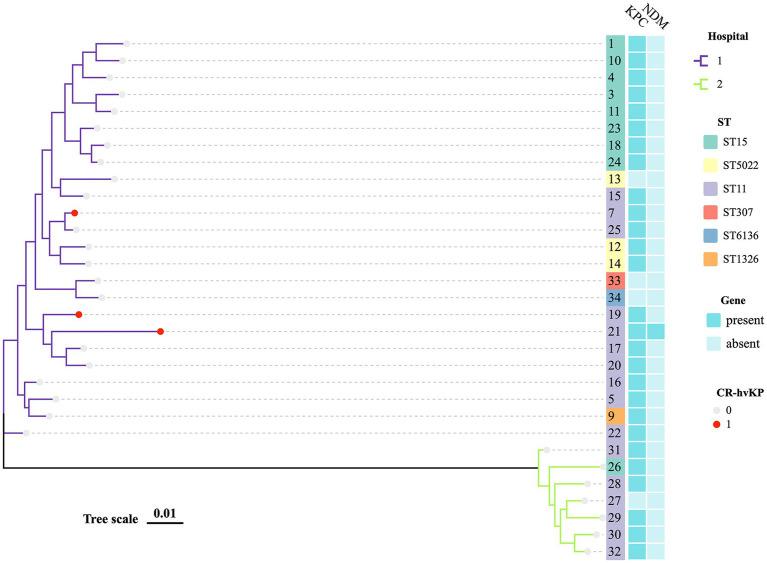
MLST phylogenetic tree of 29 CRKP and 2 CSKP. The hospital, MLST, KPC, NDM, and hypervirulent of strains in this study are highlighted. Each nucleotide location is represented by 0.01 mutations along the scale bar.

The 33 *K. pneumoniae* strains had six distinct STs and one novel ST. ST11 (16, 48.48%; 3/33 were CR-hvKP) was the most prevalent ST, followed by ST15 (9, 27.27%) and ST5022 (3, 9.09%). These STs accounted for 84.85% (28/33) of the total strains. There were also other sequence types found, but they tended to be region-specific. Besides, we discovered a rare ST307 CSKP. Strains from the same ST typically shared similar antimicrobial resistance profiles (Supplementary Tables S1, S3; Figure 1).

Of all the isolates, KPC was detected in 28 (90.32%) out of 31 isolates and was the most prevalent carbapenemase. The next-most prevalent enzyme was CTX-M (6, 19.35%) and one isolate (3.23%) carried both *KPC-2* and *NDM-6*. 19.35% of the isolates (6/31) co-produced KPC and CTX-M-lactamases, while the majority of the isolates (77.42%, 24/31) had remarkable levels of resistance to carbapenems (MIC > 32 mg/L). While DHA accounted for 71.43% in Hospital 2, it was not detected in Hospital 1 (Supplementary Table S1).

**Figure 2:**
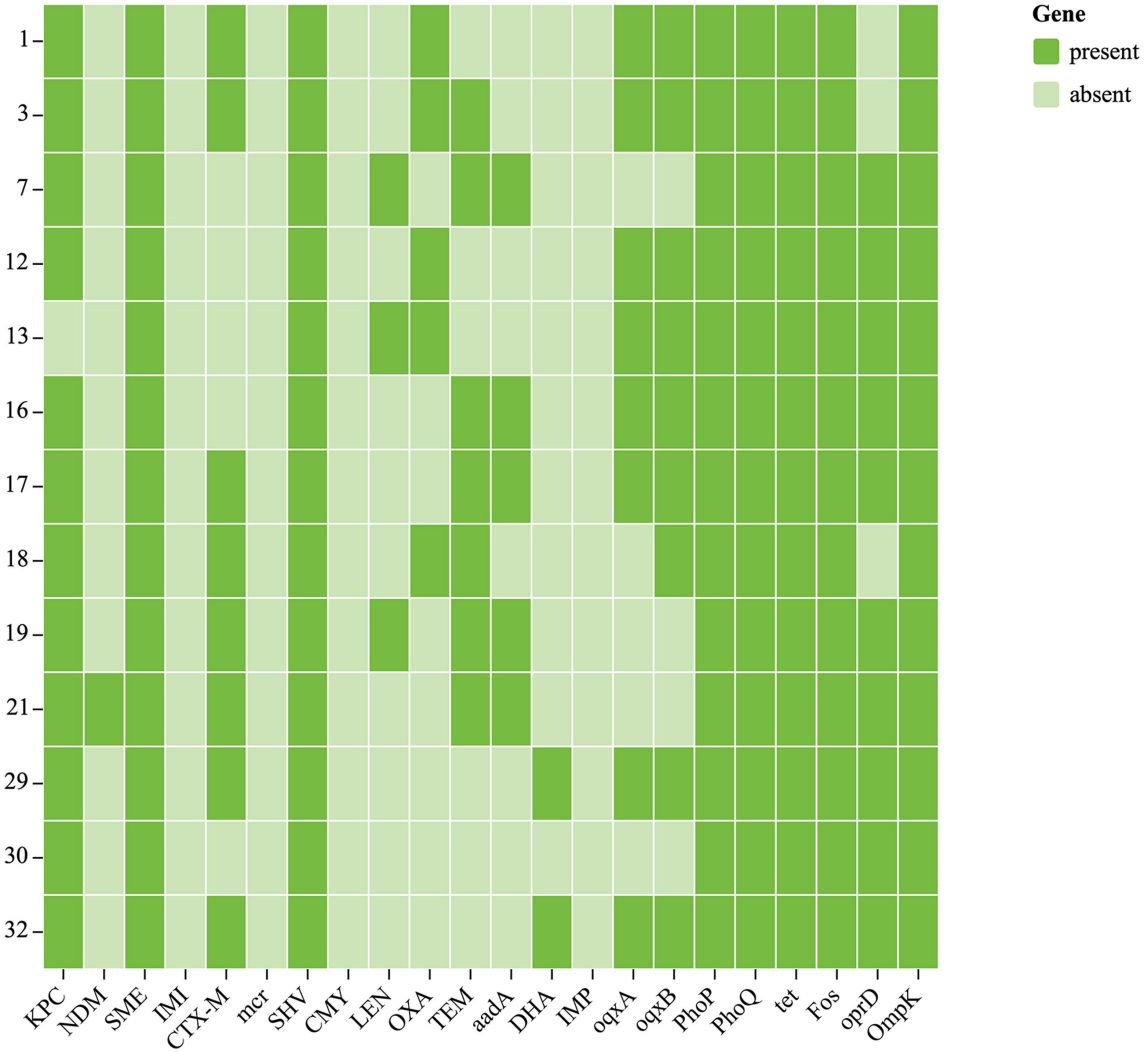
Antibiotic resistance genes of 13 CRKP. Each row denotes a strain, and each column is a gene for antibiotic resistance.

Chloramphenicol and fosfomycin resistance rates were 100%. According to WGS and bioinformatics analysis, all 13 CRKP isolates carried *SHV*, *cmlv,* and *FosA6* genes. Moreover, nearly all classes of genes conferring resistance to aminoglycosides, carbapenem, cephalosporin, fluoroquinolone, glycopeptide, macrolide, and sulfonamide were present in these CRKPs (Supplementary Table S1; Figure 2).

38.46% (5/13) of clinical isolates had CRISPR-Cas systems. Three strains were ST15 and the other two strains were ST11. All five isolates were carrying *KPC-2*. These results suggested that *KPC-2* gene could co-exist with the CRISPR-Cas systems in ST15 and ST11 CRKP.

Our results showed that 29/31 and 3/31 isolates were positive for mCIM and eCIM, respectively. Three isolates tested negative by PCR but positive in mCIM and eCIM. Further research on CRKP infections can be supported by these genetic traits, which also serve as a solid microbiological foundation (Supplementary Table S1).

### Phylogenetic analysis of strains

An MLST-based phylogenic tree showed a structure dominated by two main genetically diverse lineages that were consistent with their geographic origin. The analysis of CRKP revealed an association between the main tree branches and the ST of CRKP. There were multiple strains producing various carbapenemases in two major related clusters that belonged to various STs (Figure 1).

**Figure 3:**
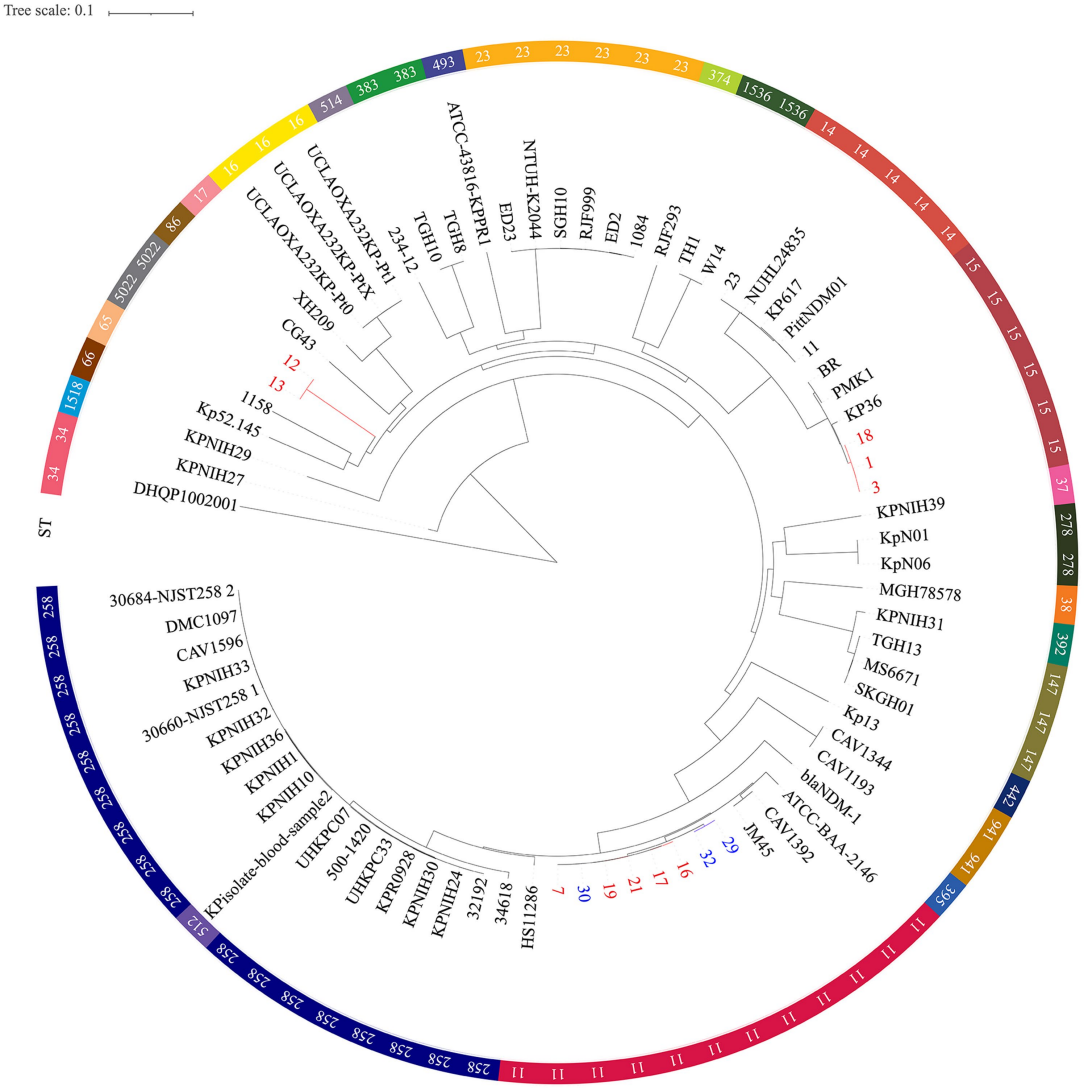
Single nucleotide polymorphism analysis of the phylogenetic tree. Circles outside of the tree indicate the STs. Different STs and locations are marked with different colors. Red, hospital 1; Blue, hospital 2; Black, other regions. The scale bar represents 0.1 mutations per nucleotide position.

Moreover, phylogenetic trees based on whole genome SNP analysis were constructed using 65 *K. pneumoniae* strains with complete genomes that were retrieved from NCBI (Figure 3). Different clades had isolates from various geographical regions and STs. Eight ST11 strains grouped together and were phylogenetically close to another ST258 cluster. Figure 3 shows that all ST11 CRKPs were grouped together with the ST11 strain hypervirulent-JM45 (NC_022082), CAV1392 (NZ_CP011578), and ATCC-BAA-2146 (NZ_CP006659). The ST15-K12 and ST11-K64 strains seem to have diverged into two distinct branches on their own. The strains in our study were phylogenetically distant from the hypervirulent strains, NTUH-K2044 (NC_012731) and SGH10 (CP025080). The other non-type CRKP 12 and 13 strains clustered with the K2 hypervirulent Kp52. 145 strain (NZ_FO834906) and ST65 1158 strain (NZ_CP006722) (Figure 3).

**Figure 4:**
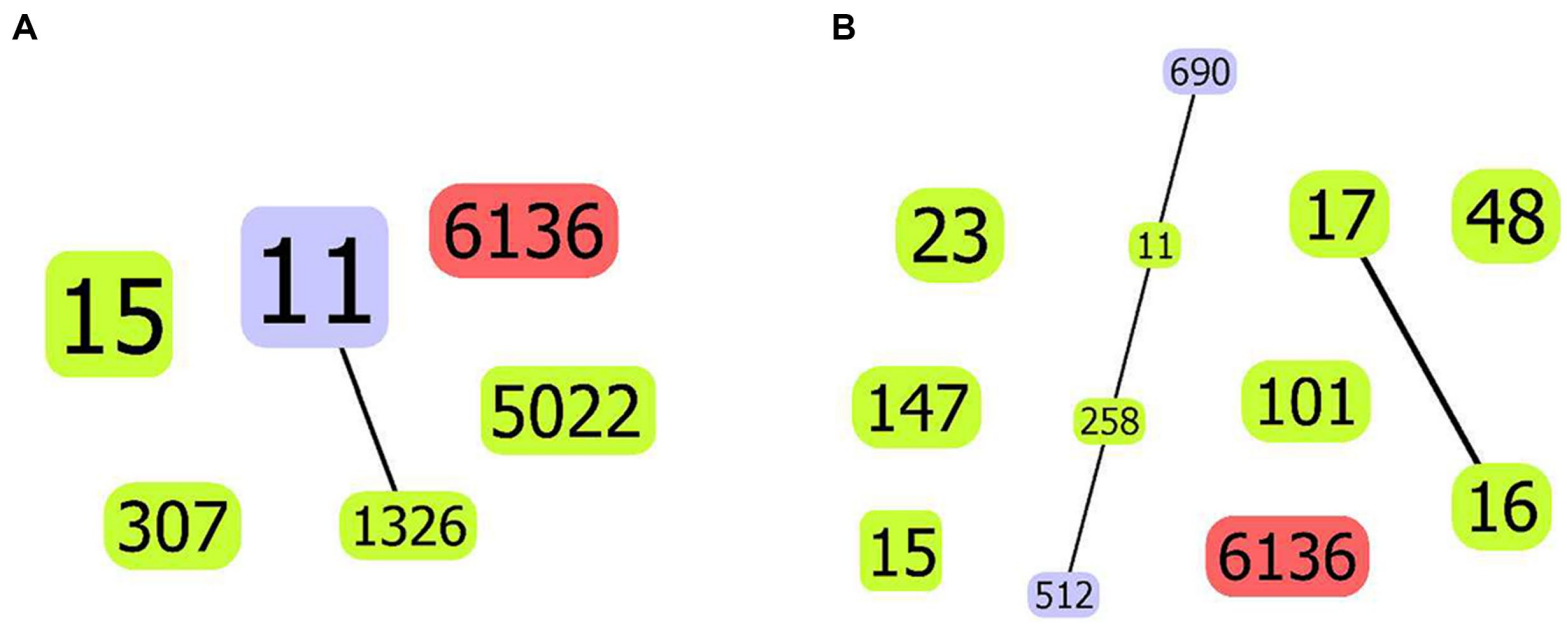
goeBurst-based population structure of ST6136 CSKP and high-risk STs. **(A)** ST6136 CSKP and high-risk STs in this study. **(B)** ST6136 CSKP and global high-risk STs. The isolated STs are shown by the blue circle. One or two different ST11 loci are shown by the purple circle, whereas the new ST is indicated by the red circle. The connections between them are shown by the black lines.

The novel ST6136 was first discovered in *K. pneumoniae*. The novel ST6136 strain was isolated from the sputum of an 87-year-old patient in September 2021 with pneumoniae. The novel ST6136 was found to be sensitive to carbapenem. The MLST data analysis using goeBURST showed that the novel ST6136 did not have a similar structure to that of the other strains in this study. When the allele of ST6136 was compared with high-risk clones (ST11, ST15, ST16, ST17, ST23, ST48, ST101, ST147, ST258, and ST690), the new clone was not found to be related to any of them (Supplementary Table S1 and Figure 4). According to the MLST-based phylogenic tree, the novel ST6136 clustered with ST307 CSKP strains from Hospital 1.

### Pathogenicity and virulence assay

**Figure 5:**
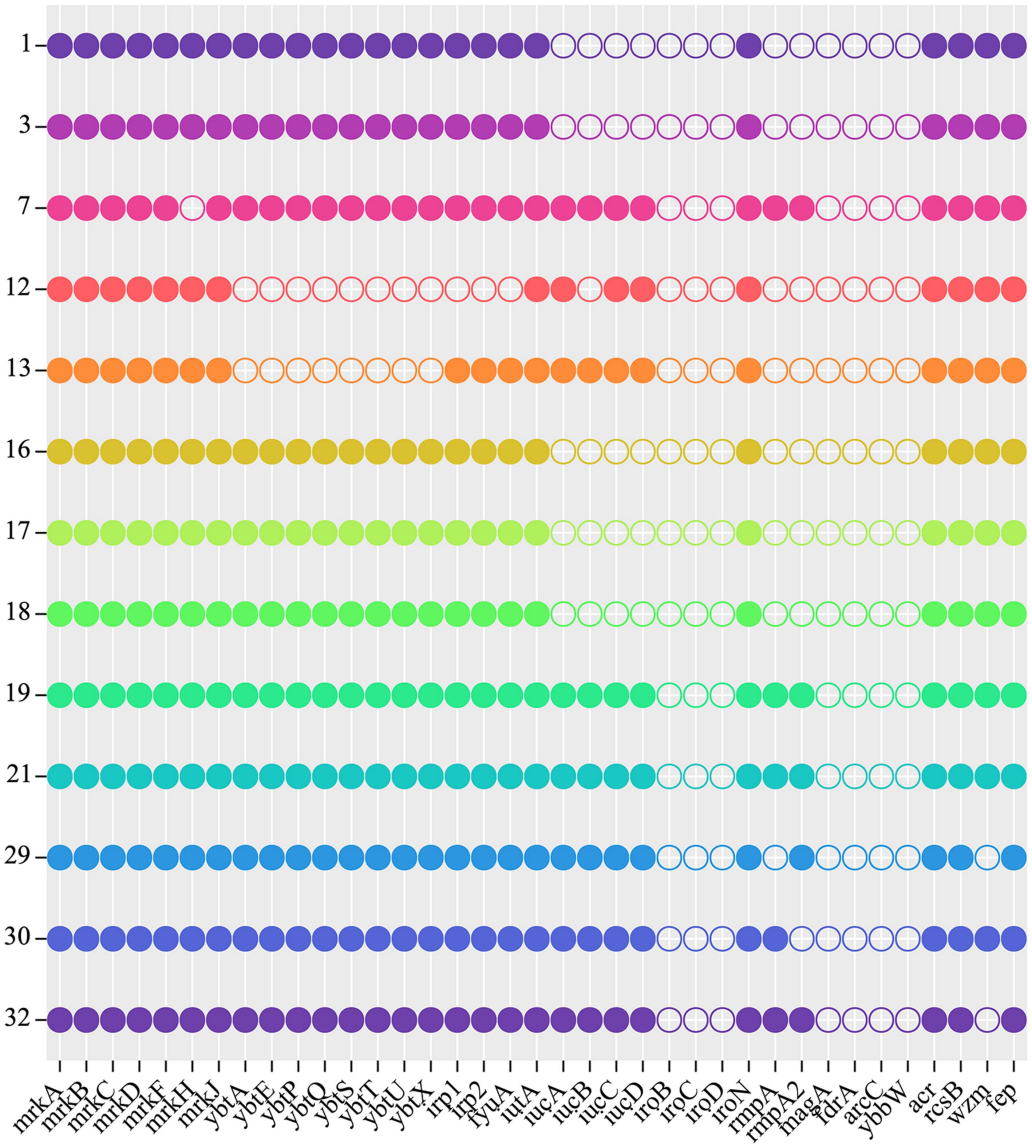
Virulence genes of 13 CRKP. Each row denotes a strain, and each column is a gene for virulence.

Nine of the total thirty-one CRKP isolates were found to be positive on the string test. The majority of these strains were serotype K64 (11/31, 35.48%) followed by K19 (10/31, 32.26%). To determine whether virulence plasmids are present, we examined the virulence gene profiles for all CRKP strains, including *iucA*, *iroN*, *rmpA*, and *rmpA2*. A positive rate of 41.94% was observed when 1–3 virulence genes were detected. The ST6136 CSKP, which was first discovered, was tested negative for all four of the examined virulence genes and was identified as KL102KL149KL155. A total of three (9.68%) out of 31 CRKP isolates were positive for all four of the tested virulence genes, suggesting the carriage of virulence plasmids (Supplementary Table S3; Figure 5). The three CR-hvKP strains were identified as ST11 K64 *KPC-2* producing *K. pneumoniae*. Of these strains, the CR-hvKP 21 strain expressed two types of resistance genes (*KPC-2* and *NDM-6*). In addition, unlike previous reports, CSKP strains carried fewer virulence genes compared to the CRKP strains (Supplementary Table S3).

**Figure 6:**
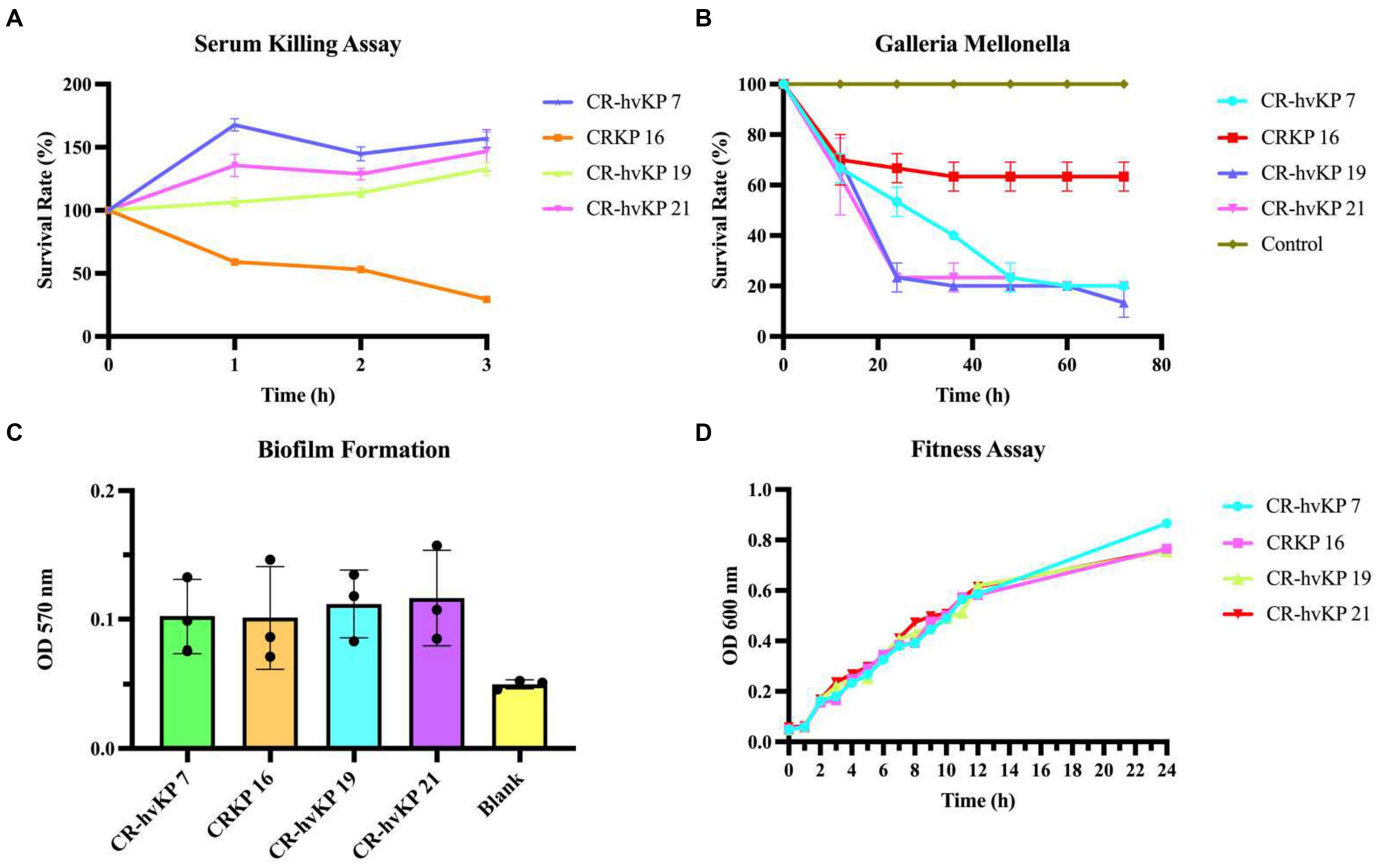
Virulence assay of three CR-hvKP. Serum killing assay **(A)**, Galleria mellonella infection model **(B)**, biofilm formation **(C)**, and fitness assay **(D)** of CR-hvKP compared with non-hypervirulent CRKP. As the negative control, CRKP-16 was a classic CRKP with lower virulence since it lacked any of the key virulence genes that were evaluated. Each strain was tested at least three times. **(A)** At 0, 1, 2, and 3 h of incubation at 37°C, 200 rpm, viable counts were evaluated. **(B)** The effect of 1 × 10^5^ colony-forming units of each *Klebsiella pneumoniae* strain on survival was assessed in *G. mellonella*. A phosphate-buffered saline injection served as the negative control group. Data are pooled from three independent experiments with 10 larvae per group per run. **(C,D)** With CR-hvKP and classic CRKP, there was no significant difference in growth or biofilm formation (*p* > 0.05).

Simultaneously, we also examined the *in vitro* and *in vivo* virulence tests among *K. pneumoniae* strains since it was inappropriate to define CR-hvKP only based on virulence genes or hypermucoviscosity. The biofilm formation test, serum killing assay, and *G. mellonella* larvae infection model were also tested to help assess strains’ virulence. CRKP-16 without all four virulence genes was used as the negative control. These CR-hvKP isolates also displayed resistance to serum killing, whereas the control CRKP-16 showed high sensitivity to serum killing (Figure 6A).

Next, using an inoculum of 1 × 10^5^ CFU, we infected the three strains with *G. mellonella* larvae. After 72 h following infection, the survival rate of ST11 CRKP-16 was 63.33%. The ST11 CR-hvKP-7, 19, and 21 resulted in 10–20% survival by 48 h, suggesting that the CR-hvKP-7, 19, and 21 strains were more virulent than CRKP-16. The consistency between the *in vitro* and *in vivo* assays supported the notion that the ST11 CRKP-7, 19, and 21, were ST11 CR-hvKP (Figure 6B). All three CR-hvKP strains and the control strain CRKP-16 formed biofilms. Moreover, the three CR-hvKP strains and the control strain CRKP-16 were classified as weak producers (Figure 6C).

### Fitness analysis

Three CR-hvKP isolates and one standard ST11 CRKP-16 were used in a growth curve experiment to perform an in-depth examination of the fitness characteristics and the danger of widespread transmission of CR-hvKP. Importantly, no significant difference between the hypervirulent strains and control strain 16 was discovered (*p* = 0.9937). This result revealed that CR-hvKP had a low fitness cost and a possible chance of spreading (Figure 6D).

### Detailed plasmid analysis of the ST11-K64 CR-hvKP strains

**Figure 7:**
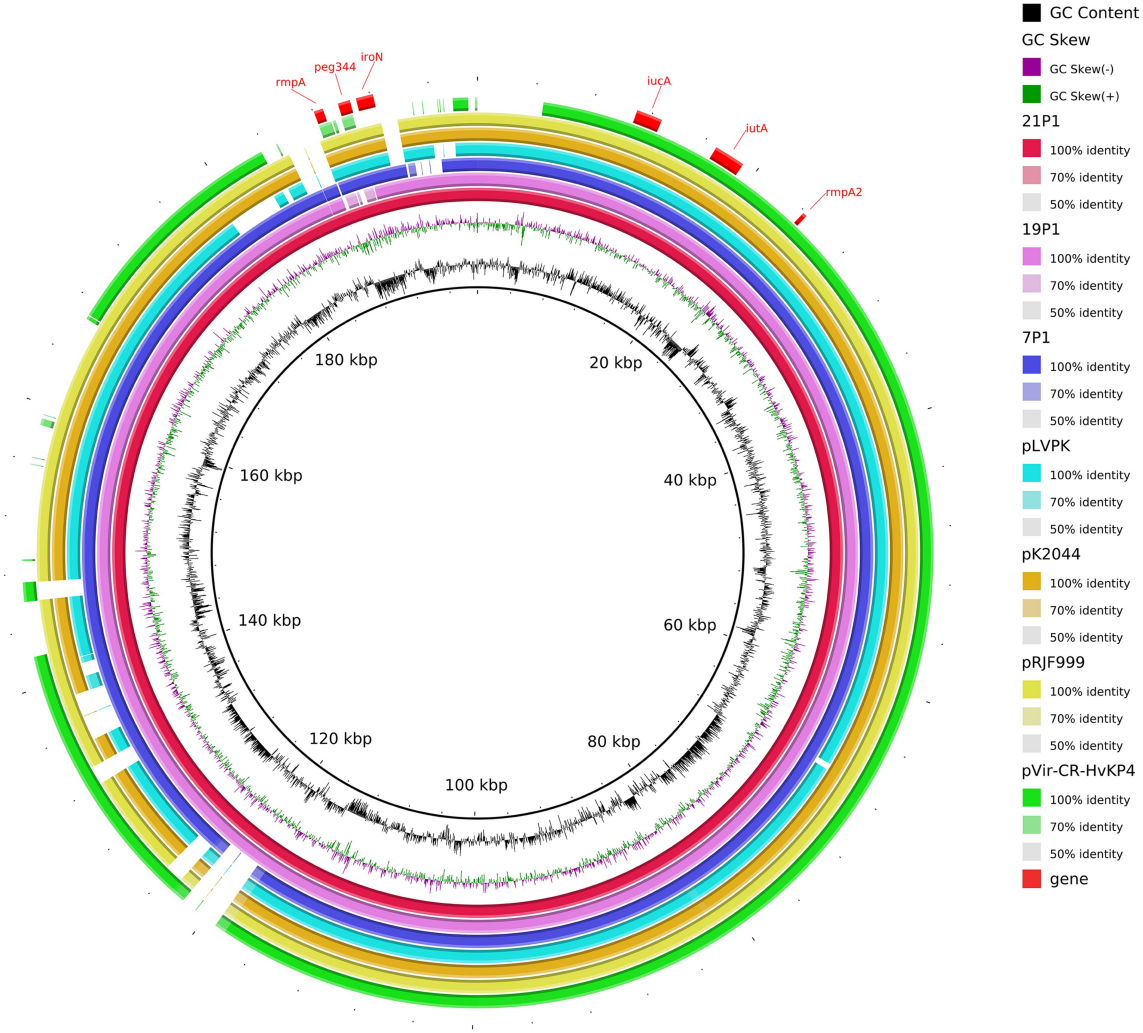
Gene map of the virulence plasmid harbored by three CR-hvKP and a comparative genomic analysis of the virulence plasmid with previously reported virulence plasmids. The GC content and GC skew are indicated from the inside out. Genes associated with hypervirulence are shown.

We inferred that these K64-ST11 CR-hvKP strains might share a virulence plasmid that was distinct from that in K47-ST11 CR-hvKP, which would be in contrast to other reports on ST11 CR-hvKP infections in China (pVir-CR-hvKP 4; Gu et al., 2018). The CR-hvKP plasmid characteristics were identified using WGS. Sorting the plasmids by size revealed a greater variety of virulence plasmids in ST11-KL64. Strain 7 contained five plasmids. Plasmid p1-CR-hvKP 7 (217,935 bp) harbored *rmpA*, *rmpA2*, *iroN*, *iucA*, and *peg344*, which is considered to be a virulence plasmid. A plasmid alignment map of p1-CR-hvKP 7, p1-CR-hvKP 19, and p1-CR-hvKP 21 with pK2044, pRJF999, pVir-CR-HvKP 4, and pLVPK showed a greater similarity for most of the genomic content and backbone (Figure 7). Aside from virulence characteristics, no resistance genes and type IV secretion system (T4SS) apparatus have been discovered in these virulence plasmids.

The plasmids p2 of the three CR-hvKP were identified as IncFII (pHN7A8) which carried the resistance genes, including the carbapenemase gene *KPC-2*, aminoglycoside resistance gene *rmtB*, beta-lactamase genes *bla*_CTX-M-65_ and *bla*_SHV-64_, TEM family class A β-lactamase ( *bla*_TEM-1_), and *mexN* among others. Unexpectedly, *NDM-6* of CR-hvKP 21 strain was located on the chromosome. In addition, the *KPC-2*-carrying plasmids of all three CR-hvKP strains were found to have the T4SS system, which mediates plasmid conjugation transfer.

These findings suggest that a combination of resistance and virulence genes carried by different plasmids contributed to the hypervirulent and multi-drug resistance of *K. pneumoniae*. Overall, the evolution of the CR-hvKP strains may be largely influenced by the virulence and resistance genes carried by plasmids.

### Plasmid comparison

**Figure 8:**
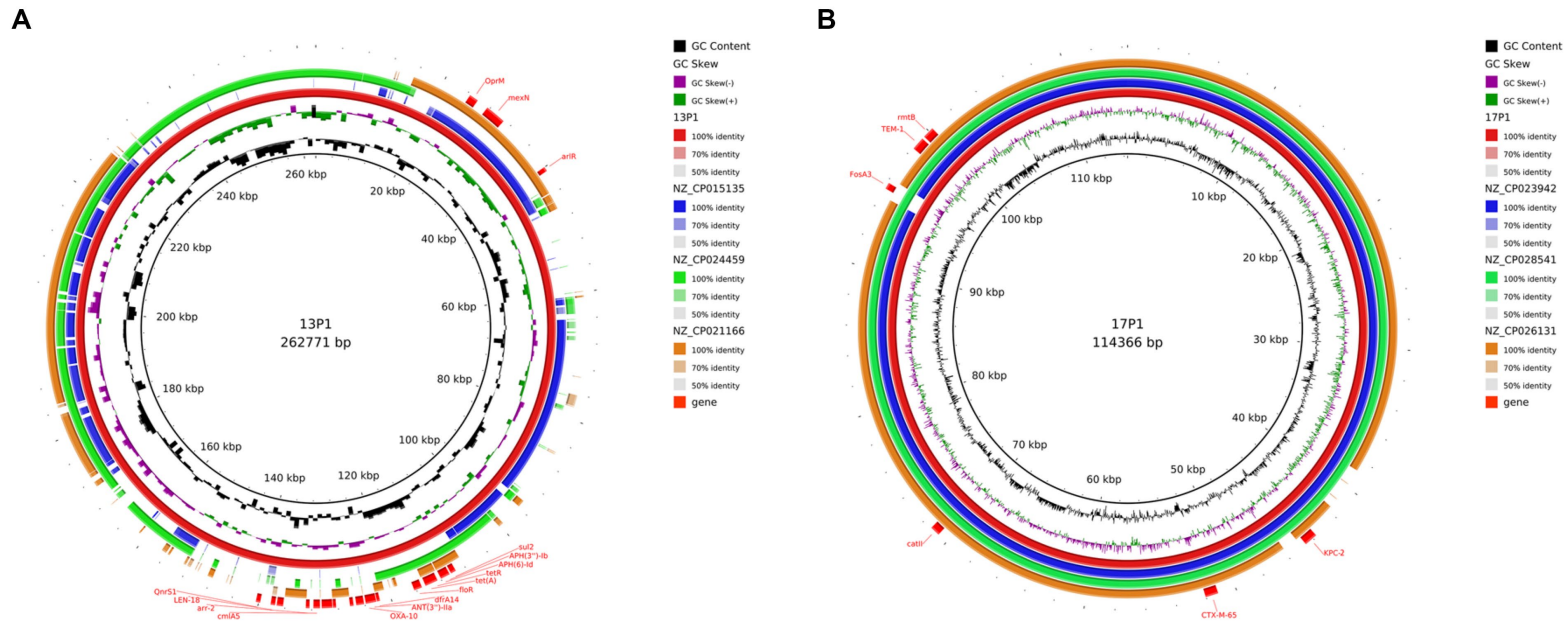
Genomic structure and comparative genomic analysis of the representative plasmid in CRKP strains. GC content and GC skew are indicated from the inside out. Genes associated with antimicrobial resistance are shown. **(A)** Plasmid 1 from CRKP 13; and **(B)** Plasmid 1 from CRKP 17.

Six distinct plasmids in all were found in the seven isolates. The WGS of seven ST11 CRKP strains showed that ST11 strains contained 2–5 plasmids varying in size from 5,596 to 217,935 bp. These isolates demonstrated the diversity of the ST11 plasmids by including a number of genes linked to virulence and resistance. The PacBio-sequenced isolates mainly had one or more plasmids harboring genes implicated in resistance to a variety of antibiotic classes (e.g., *β*-lactams, aminoglycosides, sulfonamides, and tetracycline). A search of additional resistance genes of p1-CRKP-13 revealed that resistance genes, including *arlR*, *cmlA5*, *OXA-10*, *tet(A)*, *QnrS1*, and *Sul2*, were identified in this IncFIB(K)/IncFII(K) plasmid (Figure 8A). Another plasmid, p1-CRKP-17, was classified as the IncFII-family pHN7A8 by replicon sequence typing and carried the *KPC-2*, *bla*_TEM-1_, *bla*_CTX-M-65_, *fosA3*, *catII* and *rmtB* genes. To the best of our knowledge, this is the first report of plasmid isolation from clinical patients in China.

The plasmids of our strains were compared with the plasmids in the NCBI database. The three most similar plasmids were selected for further analysis (Figure 8). In addition, those that resembled plasmids were carried by *K. pneumoniae*, *E. coli*, and *Pseudomonas aeruginosa*. This suggests that there is host diversity in these plasmids.

### Transmission of *KPC-2* conjugative plasmids

According to the WGS result, the five *KPC-2*-producing ST11 CRKP strains were found to have the T4SS system, which mediates plasmid conjugation transfer. Therefore, to test if the *KPC-2*-carrying plasmids were able to be conjugated to other isolates, conjugation tests were carried out utilizing five *KPC-2*-producing ST11 CRKP as donors and *E. coil* EC600 as a recipient. Our data showed that all the *KPC-2*-carrying plasmids could be conjugated to *E. coil* EC600. The variation in transconjugant susceptibility profiles is shown in Table 1. Transconjugants of these strains exhibited resistance to ceftriaxone and carbapenem and were found to carry the *KPC-2* gene.

**Table 1:** Conjugative transfer of carbapenem-resistant *K. pneumoniae*.

Strain ID	ST	Carbapenemase	MIC (μg/mL)			
			IPM	MEM	ETM	CRO
CRKP (donors)						
7	ST11	*KPC-2*	256	256	256	256
16	ST11	*KPC-2*	128	64	256	256
17	ST11	*KPC-2*	128	64	256	256
19	ST11	*KPC-2*	256	128	256	256
21	ST11	*KPC-2*	256	256	256	256
*E. coil* EC600 (recipient)			≤0.5	≤0.5	≤0.5	≤1
Transconjugants						
7-TC	—	*KPC-2*	64	32	64	128
16-TC	—	*KPC-2*	32	64	64	128
17-TC	—	*KPC-2*	32	64	64	128
19-TC	—	*KPC-2*	64	64	64	128
21-TC	—	*KPC-2*	32	64	128	128

CRKP, carbapenem-resistant *K. pneumoniae*; IMP, imipenem; MEM, meropenem; ETP, ertapenem; CRO, ceftriaxone.

### Antibacterial activities of Fe_2_C NPs *in vitro*

**Figure 9:**
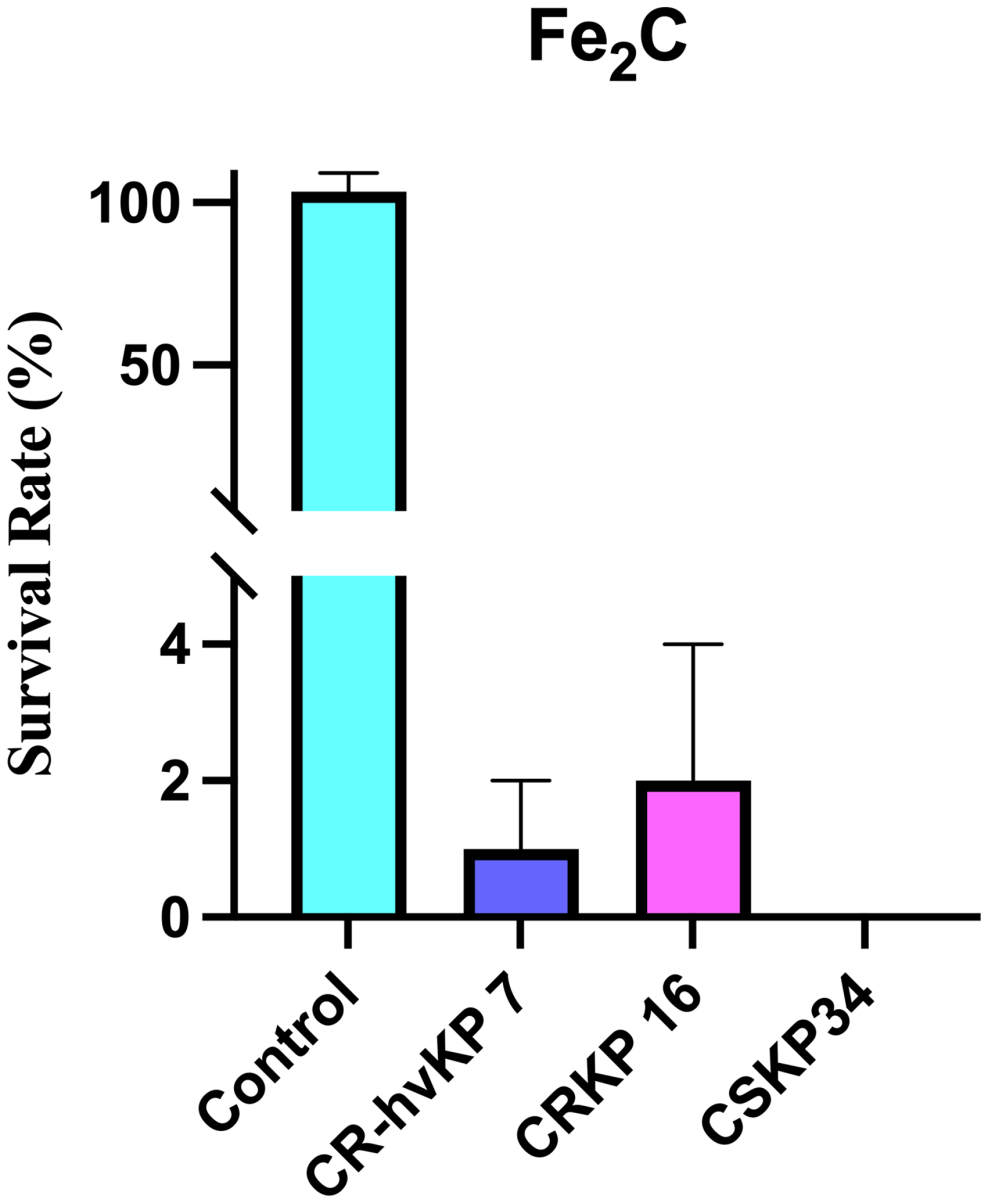
The survival rate of CSKP, CRKP, and CR-hvKP after 3 h of treatment with 75 μg/mL Fe_2_C.

The *in vitro* antibacterial activity of Fe_2_C NPs was evaluated on CSKP, CRKP, and CR-hvKP in our study. As revealed in Figure 9, Fe_2_C showed excellent inhibition against CSKP, CRKP, and CR-hvKP. These results indicate that Fe_2_C exhibited superior bactericidal properties against these strains.

## Discussion

We described the epidemiology of CRKP from two hospitals in Jiangsu province of China from November 2020 to December 2021. Also, we identified the underlying mechanisms of the MDR and virulence of *K. pneumoniae* strains. We first discovered an IncFII-family pHN7A8 isolated from CRKP carrying the *KPC-2*, *bla*_TEM-1_, *bla*_CTX-M-65_, *fosA3*, and *rmtB* genes. *KPC-2* can be transferred horizontally to *E. coil* by conjugation. The CRISPR-Cas systems in ST15 and ST11 CRKP could coexist with the *KPC-2*. Importantly, we identified an increased incidence, biofilm production, and non-inferior fitness of *KPC-2*-producing K64 CR-hvKPs. The acquisition of a plasmid with the carbapenem-resistance gene *KPC-2* in HvKP could be the reason for CR-hvKPs. Besides, a CR-hvKP was first identified as carrying the *KPC-2*, *NDM-6*, *SHV-182*, *SHV-64*, and *bla*_CTX-M-122_ genes. Thus, our findings demonstrate the importance of infection control and possibly outbreak prevention for CR-hvKPs and CRKPs, which produce *KPC-2*. Another important finding was the novel ST6136 and KL102KL149KL155 CSKP, which was negative for all four virulence genes and unrelated to high-risk clones (ST11, ST15, ST16, ST17, ST23, ST48, ST101, ST147, ST258, and ST690). In addition, our results demonstrated that Fe_2_C could effectively inhibit both CRKP and CSKP bacterial growth *in vitro*. Therefore, with further development and optimization, Fe_2_C has great potential as a new class of antibacterial agents to combat antibiotic-resistant pathogens.

The majority of our strains were ST11 clones that produced *KPC-2*, which was consistent with earlier studies (Mathers et al., 2015; Bush and Bradford, 2020; Zhu J. et al., 2020). However, our results differed from another study from Taiyuan, China. According to their research, NDM-1 was the main cause of carbapenem resistance (Zhu J. et al., 2020). In this study, we discovered one CR-hvKP strain co-producing *KPC-2*, *NDM-6*, *SHV-182*, *SHV-64*, and *CTX-M-122* genes. Especially, the CR-hvKP isolate with these genes has not before been reported internationally. Our data raise a concern about *KPC-2*, *NDM-6*, *SHV-182, SHV-64*, and *CTX-M-122* CR-hvKP, particularly in light of the widespread use of ceftazidime/avibactam, which could give the CR-hvKP a selective advantage. The spread of these strains should be closely monitored throughout the globe.

According to a previous study, compared to isolates without the CRISPR-Cas system, CRISPR-carrying isolates were more sensitive to carbapenems (Hu et al., 2023). But in our study, two *KPC-2* isolates were ST11, and three were ST15 among CRISPR-positive CRKP. One ST15 *KPC-2*-producing CRKP-18 strain carrying a *bla*_OXA-1_, *bla*_TEM-1_, *bla*_CTX-M-15_, *AAC(6′)-Ib-cr, mexN,* and *oprA*-bearing plasmids. It appears that CRISPR-Cas systems could not prevent the entrance of such resistant plasmids into ST15 isolates. Additionally, a prior study revealed that CRISPR-Cas immunity is not completely unaffected (Jiang et al., 2013). It slows down plasmid reception but does not stop it from being transferred and established. Our findings suggested that the CRISPR-Cas systems in ST15 and ST11 CRKP could coexist with the *KPC-2* gene.

*Klebsiella pneumoniae* infections are led on by various clones that are broadly dispersed geographically (Chen et al., 2014). In our study, ST11 accounts for 48.40%. Our results were in line with this finding, and 64.2% of all CRKP strains throughout the time in China were of the ST11 (Wang et al., 2018; Liao et al., 2020). Notably, over time, a marked rise in the frequency of ST15 CRKP strains was discovered (Wyres et al., 2020a), which was 27.3% in this study. In South and Southeast Asia, ST15 is responsible for the majority of CRKP cases (Wyres et al., 2020b). Besides, the novel ST6136 clustered with ST307. It calls for the adoption of more efficient infection prevention and control procedures and underlines the need of genomic surveillance for emerging novel sequence types of *K. pneumoniae*.

It has been well established that virulence and antibiotic resistance genes may be transferred horizontally between highly contagious clones (Sun et al., 2019; van Dorp et al., 2019; Yang et al., 2021). The spread of KPC has been primarily associated with transmissible plasmids, which belong to different incompatibility groups (e.g., IncFII, IncI2, IncX, IncA/C, IncR, IncN, and ColE) (Chen et al., 2014). Conjugation experiments results were in line with this finding as most of our KPC gene was carried by IncFII (pHN7A8). To our knowledge, IncFII (pHN7A8) carrying *bla*_CTX-M-65_, *fosA3*, and *rmtB* isolated from an *E. coli* from a dog in China was first reported in 2013 (He et al., 2013). An earlier investigation revealed the clonal spread of *K. pneumoniae* CG258 strains in various Chinese hospitals, which possess hybrid plasmids from the IncFII family p*KPC*-LK30/pHN7A8 that carry the *KPC-2* and *rmtB* genes (Shi et al., 2018). Importantly, we found that a conjugative plasmid pHN7A8 isolated from CRKP carried the *KPC-2*, *bla*_TEM-1_, *bla*_CTX-M-65_, *fosA3*, *catII,* and *rmtB* genes, which conferred resistance to multiple drugs. The discovery of this multidrug resistance conjugative plasmid presents further challenges to the prevention and treatment of CRKP infections. These results further suggested the zoonotic potential of IncFII-family pKPC/pHN7A8 hybrid plasmids between animals and humans.

In this study, CR-hvKPs coharboured a virulent plasmid and a *KPC-2*-carrying plasmid. Plasmid conjugation is mediated by the T4SS system (Cabezón et al., 2015; Huang et al., 2021). The T4SS structure helps to improve the adaptability of bacteria to the external environment, strengthen the existence of drug-resistant bacteria, and accelerate the spread of drug resistance (Zhu W. et al., 2020; Zeng et al., 2021). According to the results of WGS on plasmids, the T4SS system was found in IncFII-family pKPC/pHN7A8 hybrid plasmids, but not in IncR virulence plasmids. Conjugation experiments suggested that *KPC-2* can be transferred horizontally between other species. These results further suggested the mechanism for widespread CR-hvKP could be the transfer of conjugative plasmid pKPC/pHN7A8 into HvKP. The ST11 strain is the most common CRKP in China, hence the co-existence of virulence and resistance plasmids in these strains is highly alarming. The development of CRKP as a causal pathogen raises serious issues for public health.

We recovered three *KPC-2* producing ST11-K64 CR-hvKP isolates from patients in Jiangsu. The virulence plasmids in the *KPC-2* ST11 clone, which is the most prevalent CRKP in China, are extremely concerning. We identified the genes *iucA*, *iroN*, *rmpA*, *rmpA2*, and *peg344* in the virulence plasmid of CR-hvKP. The gene *peg-344* was considered a biomarker for hvKP in a recent study (Russo et al., 2018) and our results also support this finding. In addition, in tests using serum killing assays and *G. mellonella* infection assays, we discovered that strains with CPS regulator genes (*rmpA* or *rmpA2*), salmochelin (*iroBCDN*), and aerocin (*iucABCD*) siderophore gene clusters exhibited greater levels of virulence. This finding supports the idea that these genes are crucial for *K. pneumoniae’s* virulence phenotype (Gu et al., 2018; Yang et al., 2020; Zhang et al., 2020). Moreover, CR-hvKP 19 was found to be phylogenetically more closely related to CR-hvKP 21 than CR-hvKP 7. However, the isolation time of the CR-hvKP 19 and CR-hvKP 21 strains differed by only 6 months, suggesting that *KPC-2* CR-hvKP could spread and exist stably in the clinical environment. Such strains may cause serious and incurable illnesses. Of note, an organism frequently incurs a large fitness cost due to increased virulence or drug resistance (Yong et al., 2022). Unexpectedly, our data also show that because of low fitness costs, the spread of hypervirulence might occur very speedily. Furthermore, the cell membrane structures may enhance *K. pneumoniae* colonization and persistence *in vivo* by enabling contact with host tissues, effective outgrowth, and biofilm formation (Wang et al., 2020). All three *KPC-2* and KL64 CR-hvKP strains investigated in this study can generate biofilms, suggesting that we should be vigilant about the colonization and infection ability of such strains. Finally, these results highlight the importance of improved tracking and control of such organisms.

Fe-based nanomaterials exhibit a superior magnetothermal effect in an alternating magnetic field, as well as superior peroxidase-like properties as nanozymes, able to catalyze H_2_O_2_ to create ·OH radicals in a region where bacteria are infected and there is a low pH and a huge amount of H_2_O_2_ (Sun et al., 2021). The bactericidal mechanisms of nanometallic materials have been shown to be related to the release of metal ions and the accumulation of reactive oxygen species (ROS) (Wang et al., 2022). The present study demonstrates the promising potential of the use of a thermogenic nanozyme as a synergistic antimicrobial platform.

It could be more reasonable to characterize the hvKP strains by combining bacterial and clinical characteristics. Here, rather than clinical characteristics, laboratory-based tests were used to define a hypervirulent strain. Hence, more research including genetics, phenotypic, and clinical factors may help to better characterize the hvKP strains. Moreover, a more in-depth understanding of the evolutionary capacity by which *KPC-2* produces ST11-K64 CR-hvKP to become increasingly pathogenic is vital to identify, keep track of, and create measures that will reduce the likelihood that such pathogens will have a negative effect on human health.

## Conclusion

The prevalent CRKP of the two hospitals was ST11-K64 *KPC-2*-producer. We first reported a dangerous CR-hvKP, which was a two-carbapenemase producer: *KPC-2*, *NDM-6*, *SHV-182*, *SHV-64,* and *bla*_CTX-M-122_. Ceftazidime/avibactam’s broad use could give this CR-hvKP a selective advantage. Our findings also demonstrate that hypervirulence dissemination in ST11 *KPC-2* CR-hvKP could be extremely rapid due to limited fitness costs. Besides, a conjugative pHN7A8 plasmid carrying the *KPC-2*, *bla*_TEM-1_, *bla*_CTX-M-65_, *fosA3*, *catII,* and *rmtB* genes was discovered to show resistance to multiple drugs and have zoonotic potential. Furthermore, five ST15 and ST11 CRKPs had CRISPR-Cas systems. The novel ST6136 CSKP without hypervirulence was first discovered and not related to selected high-risk clones. Finally, our novel Fe_2_C nanomaterials will contribute to the prevention and therapy of CSKP, CRKP, and CR-hvKP infections. Our findings will help to further address the growing incidence of multidrug-resistant nosocomial infections and provide patients with the best care possible in the future.

## Data availability statement

The datasets presented in this study can be found in online repositories. The names of the repository/repositories and accession number(s) can be found in the article/Supplementary material.

## Author contributions

JZ, JD, and XKZ conceived and designed the research. YJ, XYZ, and TC conducted experiments and analyzed the data. JZ wrote and revised the manuscript. All authors read and approved the final manuscript.

## Funding

This study was funded by the National Natural Science Foundation of China (grant no. 32172855). The funders had no influence in the design of the study, data collecting and analysis, or the decision to publish the study.

## Conflict of interest

The authors declare that the research was conducted in the absence of any commercial or financial relationships that could be construed as a potential conflict of interest.

## Publisher’s note

All claims expressed in this article are solely those of the authors and do not necessarily represent those of their affiliated organizations, or those of the publisher, the editors and the reviewers. Any product that may be evaluated in this article, or claim that may be made by its manufacturer, is not guaranteed or endorsed by the publisher.

## Footnotes

^1^http://bigsdb.Pasteur.fr/klebsiella/

^2^https://itol.embl.de/
